# Family and Developmental History of Individuals With Autism Spectrum Disorder: Importance of the Clinical Diagnostic Interview for Diagnosis in Adolescents. An Explorative Study

**DOI:** 10.3389/fpsyt.2021.703023

**Published:** 2021-10-26

**Authors:** Johanna Waltereit, Charlotte Czieschnek, Katja Albertowski, Veit Roessner, Robert Waltereit

**Affiliations:** ^1^Department of Child and Adolescent Psychiatry, Medical Faculty Carl Gustav Carus, Technische Universität Dresden, Dresden, Germany; ^2^Department of Child and Adolescent Psychiatry, University Medical Center Göttingen, Göttingen, Germany

**Keywords:** autism spectrum disorder, late diagnosis, family history, developmental history, adolescents, clinical diagnostic interview

## Abstract

**Background:** Diagnosis of autism spectrum disorder (ASD) can be made early in childhood, but also later in adolescence or adulthood. In the latter cases, concerns about an individual's behavior typically lead to consultation of a mental health professional (MHP). As part of the initial clinical examination by the MHP, a clinical diagnostic interview is performed, in order to obtain the patient's history, and may lead to the hypothesis of ASD. We were here interested to study family and developmental history as key parts of the patient's history. The aim of the study was to investigate empirical differences between adolescents with ASD and adolescent control persons in family and developmental history.

**Method:** Clinical diagnostic interview items addressing family and developmental history were adopted from their regular use at several university hospitals and in leading textbooks. Parents of male adolescents with normal intelligence and an ASD diagnosis (*n* = 67) and parents of male adolescents without psychiatric diagnosis (*n* = 51) between the age of 12 and 17 years were investigated. Data were operationalized into three categories: 0 = normal behavior, 1 = minor pathological behavior, and 2 = major pathological behavior. Differences were analyzed by multiple *t*-test of two-way ANOVA.

**Results:** Adolescents with ASD expressed a profile of items significantly differing from control persons. Comparison of significant items with the empirical ASD literature indicated robust accordance.

**Conclusions:** Our findings support the importance and feasibility of the clinical diagnostic interview of family and developmental history for initiation of the diagnostic process of ASD in adolescents.

## Introduction

Autism spectrum disorders (ASDs) are a group of childhood-onset neurodevelopmental disorders characterized by several core symptoms including enduring impairment of social communication and interaction that occur across multiple contexts. Additionally, restricted, repetitive behaviors, and interests or activities as well as sensory symptoms can be found. ASD is a diagnostic category in the American Psychiatric Association's *Diagnostic and Statistical Manual of Mental Disorders*, 5th edition (1). The WHO International Classification of Diseases, 10th edition (ICD-10) (2), describes the following diagnostic categories: autism, Asperger's syndrome, and pervasive developmental disorder not otherwise specified. ASD are currently conceptualized by most researchers as a continuum of the same disorder with varying degrees of severity, associated intellectual functioning, and medical conditions (3). Recent studies estimated the prevalence of ASD to around 0.5–1% (4, 5). ASD is predominantly genetically determined with a heritability of ~90% (6).

The hypothesis-based diagnostic procedure at specialized ASD centers or teams is well established and equipped with ASD-tailored instruments, notably the gold standards Autism Diagnostic Interview—Revised (ADI-R) (7) and Autism Diagnostic Observation Schedule (ADOS) (8). However, much earlier in this diagnostic process, when there is no hypothesis yet of ASD, there are concerns about the child's, the adolescent's, or even the adult's behavior. In the following, we describe diagnostic pathways that are established but not standardized in Germany (9, 10) and may reflect the situation in other European countries as well, where the healthcare system divides into general practitioners and specialists like mental health professionals (MHPs). It should be kept in mind that healthcare systems are globally very diverse and may in other regions differ from these descriptions (11–13). In young children, the family usually consults the pediatrician or general practitioner. In older children, adolescents, or adults, an MHP may be consulted directly or by referral from other professionals. In this context, an MHP is understood as a specialist in child and adolescent psychiatry, adult psychiatry, or clinical psychology, treating patients in a general mental healthcare setting. The hypothesis of ASD made by these professionals may then lead to referral to a specialized ASD center or team, as described above, where the diagnosis of ASD can be confirmed or discarded.

The majority of ASD diagnoses are made during childhood, and much effort has been made to improve early diagnostic processes. However, time to diagnosis and diagnostic quality are not satisfactory (10, 14). A reasonable amount of ASD diagnoses are not detected before adolescence and adulthood (15). In particular, ASD is often diagnosed not before adolescence or even adulthood when accompanied by normal or high intelligence (16–18). As described above, the gatekeeper to further assessment at specialized ASD centers or teams is in this age group usually an MHP. In this study, we focus on the detection of psychopathological signs and narratives in adolescents that result in the hypothesis of ASD by MHPs and initiate specific diagnostic processes.

The psychopathological assessment by the MHP is typically tripartite including a clinical diagnostic interview, the use of general rating scales, and ideally setting-specific information like school reports (19–22) and is conducted by MHPs, as described above. The clinical diagnostic interview is a recall of memories of certain events, stored in the brains of patients and their relatives. These memories are the subjective views of patients and relatives and can differ from an objective assessment of described events. During this face-to-face-interview, patients and parents are asked questions on several domains, such as current symptoms, history of present illness, developmental history, personal history, and family history, assessed by the professional interviewer. Additionally, several structured interviews and rating scales are used for clinical assessment. For general psychopathology in children and adolescents, rating scales such as the Child Behavior Checklist (CBCL) (23), the Strengths and Difficulties Questionnaire (SDQ) (24), and the Kiddie-Sads-Present and Lifetime Version (K-SADS-PL) (25) are widely used instruments. However, these psychopathology screening tools do only touch important aspects of family and developmental history. Specific screening tools for ASD like the Social Responsiveness Scale (SRS) (26) or the Autism Spectrum Quotient (AQ) (27) are not part of the general clinical examination, as their use presupposes that there is already a hypothesis of ASD. Further, there is a fundamental “technological” difference between the clinical diagnostic interview on the one side and structured interviews and rating scales on the other side. Structured interviews and rating scales focus on obtaining a complete and specific set of data; their structuredness is at the expense of flexibility and building the relationship between a patient and an interviewer. In contrast, the clinical diagnostic interview technique is a face-to-face-interview between a patient and clinician, is part of building a professional relationship, provides maximal flexibly, and is a general tool to assess many different diagnostic hypotheses.

Family and developmental history takes an important part in the diagnosis of ASD: “Include in every autism diagnostic assessment (…) details of the child's or young person's experiences of home life, education and social care (…) a developmental history, focusing on developmental and behavioral features (…) a medical history, including prenatal, perinatal and family history, and past and current health conditions (…)” (12). The clinical diagnostic interview is as a method described in all major textbooks. However, the literature is scarce on *empirical* analyses of the family and developmental history data obtained in the clinical diagnostic interview. In a recent study, investigation of family and developmental history in attention-deficit/hyperactivity disorder (ADHD) patients revealed a significant profile in contrast to control subjects, and the profile could be demonstrated for robust accordance with the ADHD literature (28). To our knowledge, there is so far no *empirical* literature on the clinical diagnostic interview of family and developmental history, regularly performed by the MHP during the initial phase of generating an ASD hypothesis.

As indicated above, structure and exploration technique of the clinical diagnostic interview are in principle described in leading textbooks (20–22). As such, the clinical interview technique has a strong face validity among clinicians. Yet within the generally accepted canon, there is no list of concrete and detailed items to be assessed in the clinical diagnostic interview, based on empirical evidence within language areas, countries, or specialist societies. In clinical routine, the technique is used differently among individual clinicians and individual settings. To study the clinical diagnostic interview of family and developmental history in its naturalistic setting, we had created a version derived from local guidelines used at different child and adolescent psychiatry university hospitals in Germany as well as from the descriptions in leading international psychiatric textbooks (28). In the following, we will label this set of 104 items of questions on family and developmental history as “family and developmental history questionnaire.” In the naturalistic clinical setting, the MHP recognizes the answers and interprets them as age-appropriate behavior or as potentially pathological behavior. To model this step of assessment conducted by the MHP and study it quantitatively, we operationalized the mostly verbal (non-metric) data obtained with the “family and developmental history questionnaire,” into three categories (0 = normal behavior, 1 = minor pathological behavior, and 2 = major pathological behavior) (28). As family and developmental history is typically obtained from parents of patients, we assessed all data from parents of adolescents with ASD or from parents of adolescent control persons. The “family and developmental history questionnaire” was explicitly designed to study the naturalistic scenario of the initial diagnostic process by MHPs. It is not to be understood in turn as a novel structured interview by itself.

The objective of this study was to investigate with *quantitative empiricism* the clinical diagnostic interview technique focusing on family and developmental history, in the framework of a naturalistic setting modeling the ASD initial detection phase in adolescents, as performed by MHPs.

## Methods

### Parental Participants, Adolescents With Autism Spectrum Disorder, and Control Persons

In this study, we investigated family and developmental history of adolescents with ASD and of control persons without psychiatric diagnosis, by interviewing their parents. In the following, the participants are the parents.

This sample consists of two groups of participants. The first group is parents with a child diagnosed with ASD (*n* = 67), and the second group is parents with a child in the absence of any psychiatric diagnosis, counseling, or treatment, referred to as control group (*n* = 51). Other children of the same parents did not influence the attribution to the group of participants. ASD diagnoses according to ICD-10 were *Childhood Autism* (*F84.0*), 20 children (35.7%); *Atypical Autism* (*F84.1*), seven children (12.5%); *or Asperger's syndrome* (*F84.5*), 29 children (51.8%).

Adolescents with ASD had received the diagnosis at the specialized ASD center of Child and Adolescent Psychiatry, University Hospital Carl Gustav Carus Dresden. All ASD diagnoses were made according to the diagnostic criteria of ICD-10 and the German clinical guidelines for ASD (29), under the supervision of KA, who is a board-certified specialist in child and adolescent psychiatry and head of the center. Tables 1, 2 show characteristics of participants in the ASD group and of their children, including symptom severity in ADI-R and ADOS scales. The ADI-R and ADOS data confirm that the ASD group in our study was valid under research conditions.

**Table 1:** Description of the sample.

**Characteristics of adolescents and their parents**	**ASD (*****n*** **= 67)**		**Control (*****n*** **= 51)**		**Group differences**		
	***M* (*SEM*)**	**Min–max**	***M* (*SEM*)**	**Min–max**	***t* ratio**	***df***	***p***
Age at survey	15.82 (0.38)	10–26	16.06 (0.41)	12–22	0.44	100	0.663
Age at diagnosis of ASD	9.91 (0.52)	3–11					
IQ	106.05 (1.51)	86–135	110.92 (1.88)	89–124	1.92	78	0.059
**School placement**	***n***	**%**	***n***	**%**	***t ratio***	***df***	***p***
Special needs setting	41	73.21	1	2.12	3.81	101	<0.001
Middle school	24	40	15	31.91	1.03	100	0.402
High school	25	45.45	35	68.08	2.33	100	0.021
**Diagnosis (ICD-10)**	***n***	**%**					
Childhood autism (F84.0)	20	35.70					
Atypical autism (F84.1)	7	12.50					
Asperger's syndrome (F84.5)	29	51.80					
**Education of mothers**	***n***	***%***	***n***	***%***	***t ratio***	***df***	***p***
10 years of education	30	53.57	14	29.78	2.63	100	0.015
13 years of education	24	42.85	33	70.12	2.57	100	0.005
University degree	23	41.07	25	53.19	1.47	100	0.223
**Employment of mothers**							
Unemployed	6	10.71	3	6.38	0.77	99	0.443
Part-time employment	24	42.85	10	21.27	2.36	99	0.020
Full-time employment	25	44.64	33	70.21	2.73	99	0.009
**Education of fathers**							
10 years of education	28	52.83	14	30.43	2.29	97	0.024
13 years of education	25	47.16	33	69.56	2.29	97	0.024
University degree	19	35.84	27	57.44	2.19	98	0.031
**Employment of fathers**							
Unemployed	2	3.57	1	2.12	0.48	98	0.668
Part-time employment	1	1.78	4	8.51	1.52	98	0.116
Full-time employment	50	89.28	42	89.36	0.91	98	0.990

*The first group represents adolescents with a diagnosis of ASD and the other group neurotypically developing adolescents (control). Participants of the study were the parents of the adolescents from these two groups. Columns show M and detailed results from two-way ANOVA*.

*ASD, autism spectrum disorder; ICD-10, International Classification of Diseases, 10th edition*.

**Table 2:** ADI-R and ADOS measures of the adolescents with ASD.

	**Module 1 (*n* = 4)**	**Module 2 (*n* = 3)**	**Module 3 (*n* = 45)**	**Module 4 (*n* = 7)**
	***M* (*SEM*)**	***M* (*SEM*)**	***M* (*SEM*)**	***M* (*SEM*)**
**ADOS domains**				
Communication (C)	7.50 (0.25)	5.33 (0.72)	3.56 (0.26)	3.71 (1.08)
Social interaction (SI)	10.50 (1.03)	7.66 (1.44)	6.97 (0.41)	4.57 (0.67)
Total score (C+SI)	18.00 (1.22)	13.00 (2.16)	10.53 (0.59)	8.28 (1.44)
Imagination/creativity	3.75 (0.22)	1.33 (0.27)	1.41 (0.11)	1.00 (0.29)
Restricted repetitive behaviors (RRBs)	1.50 (0.96)	1.33 (0.27)	0.82 (0.15)	0.42 (0.28)
**ADI-R domains**				
Social interaction	12.25 (1.14)	12.66 (2.23)	18.39 (0.70)	16.57 (2.28)
communication	13.50 (2.41)	11.00 (2.49)	11.89 (0.61)	12.14 (2.03)
Restricted repetitive behaviors (RRBs)	3.75 (0.96)	3.66 (0.72)	3.89 (0.31)	3.28 (0.63)

*Measures were taken from the patient files and had been assessed before inclusion in the study. Columns show M and detailed results from two-way ANOVA*.

*ADI-R, Autism Diagnostic Interview—Revised; ADOS, Autism Diagnostic Observation Schedule; ASD, autism spectrum disorder*.

Control persons were adolescents selected from a community-based pool of volunteers registered for research study participation. They had never received a psychiatric or neurological diagnosis (ICD-10 chapters F and G) by their pediatrician, general practitioner, or any other healthcare professionals. The absence of a psychiatric or neurological history was checked before inclusion by the study team.

Inclusion criteria in both groups were parents of children of male gender and an age of at least 12 years. In the ASD group, diagnosis of ASD was made before the 18th birthday; in the control group, there was no psychiatric or neurological diagnosis before the 18th birthday, accordingly. As described in Table 1, ASD diagnoses were made between the age of 3 and 11. As ASD is a persistent diagnosis, it was presupposed that these individuals had an ASD diagnosis at the age of 12–17. The oldest individual with ASD was at time of inclusion in the study 26 years old; the oldest individual without psychiatric diagnosis was 23 years old. In the ASD group, one individual was included in the study 1 day before his/her 11th birthday. This single exception was based on the assumption of a persisting diagnosis of ASD at the 12th birthday. Control participants included in the study were parents of children with an IQ between 70 and 130. Parents of children with ASD and another present psychiatric disorder, or an IQ lower than 70 or higher than 135, were excluded from this study.

The study has been approved by the Ethics Committee of the University Hospital Carl Gustav Carus (reference number EK 295072016) and was performed in accordance with the ethical standards laid down in the 1964 Declaration of Helsinki and its later amendments. Participants and also their children gave written informed consent to participate.

### Recruitment

#### Recruitment of Participants

Parents of adolescents with a former or current ASD diagnosis and of adolescents without any psychiatric diagnosis were recruited as participants. Adolescents with ASD were patients at the specialized ASD center of Child and Adolescence Psychiatry, University Hospital Carl Gustav Carus, Dresden. Adolescents without any psychiatric diagnosis were selected from a community-based pool of volunteers registered for research study participation. All participants were contacted by telephone and invited for an interview. Both parents and children provided written informed consent before being included in the study.

#### Interviewers

Medical students from the Medical Faculty Carl Gustav Carus, Technische Universität Dresden, in their final year of medical studies, participated in the project as part of a voluntarily scientific program and without financial benefit. They were thoroughly trained in the “family and developmental history questionnaire” and were always supervised by a board-certified specialist in child and adolescent psychiatry. Interviewers were not blinded for the diagnosis of the patients and were equally distributed among both groups.

#### Interview

Parents and interviewers met for an agreed appointment. Family and developmental history data were collected from at least one parent. Modeling a clinical setting, a participant and an interviewer were seated face to face in an examination room of the hospital. The interviewer asked the questions in the same order as they can be found in Supplementary Table 1 and documented the answers of parents word by word.

### Measures

#### Family and Developmental History Questionnaire

The “family and developmental history questionnaire” to study family and developmental history in the clinical examination scenario by the MHP has been described (28) and consists of a selection of relevant questions that could still be answered in a 45-min interview. These questions are commonly used for diagnostics at several German academic child and adolescent psychiatry departments and were aligned with leading textbooks (20–22). Questionnaires and instructions for the clinical diagnostic interview were obtained from the child and adolescent psychiatry departments at the university hospitals Göttingen, Hamm, Leipzig, Mannheim, Tübingen, Würzburg, and Dresden. Those items concerning family and developmental history were considered. The questions and possible answers are presented in Supplementary Table 1.

#### Operationalization

Operationalization was oriented on the CASCAP-D system, with in turn was developed for children and adolescents on basis of the AMDP System for adult patients in psychiatry and the WHO ICD-10 diagnostic inventory (30). The CASCAP-D system is used to operationalize psychopathological items. These items can be categorized into not present/regular (“0”), lightly (“1”), moderately (“2”), or severely present (“3”). For the operationalization of patient history data, most items in the “family and developmental history questionnaire” were oriented on the description of deficient or pathological behavior. In this sense, these history items were categorized into regular behavior (“0”), minor pathological (“1”), and major pathological behavior (“2”). We decided for this more simple decision tree to ensure a straightforward categorization into three options: obviously neurotypical behavior, pathological behavior as described in WHO ICD-10/DSM-V or obviously (from the viewpoint of a board-certified specialist in child and adolescent psychiatry) associated with psychiatric disorder, and behavior at risk for psychiatric disorder. This more simple categorization is similar to the K-SADS structured interview (31). Some items represented different levels of social function or burden of disease rather than psychopathology. In those cases, representing markers of social or educational functioning or degree of disease burden, history items were—in analogy—categorized into high psychosocial functioning (“0”), regular psychosocial functioning (“1”), and low psychosocial functioning (“2”) or no disease burden (“0”), low disease burden (“1”), and high disease burden (“2”), respectively.

Interviewers wrote down the answers to a specific question provided by the parents. In case of non-metric answers, interviewers made a preliminary operationalization according to the rules described above. Primary data sheets with all given answers were regularly reviewed together with the interviewers during study team meetings led by a specialist in child and adolescent psychiatry (RW), and operationalization was finalized. Modeling the scenario of the clinical diagnostic interview, a regular clinical technique performed by MHPs, the study team interpreted during the process of operationalization the answer of the parents in terms of neurotypical vs. psychopathological behavior or constellation, as described above.

#### Collection of Autism Diagnostic Interview—Revised and Autism Diagnostic Observation Schedule Data

The ADI-R (7) and ADOS (8) have high accuracy as diagnostic instruments in research settings. Using the semi-structured diagnostic interview ADI-R, a trained MHP asks a parent or caregiver detailed questions about development and underlying behaviors associated with ASD. The total cutoff score of the ADI-R, to be considered positive for a diagnosis of ASD, is for the communication and language domain 8 for verbal subjects. For all subjects, the cutoff for the social interaction domain is 10, and the cutoff for restricted and repetitive behaviors is 3 (32). The ADOS is a semi-structured, 45- to 60-min observation and interaction session with an evaluator and the child, assessing social communication and interaction and playing behavior. There are four modules for different age groups. ADOS values were interpreted as suggestive of a diagnosis of ASD if the subject met the cutoff values in the communication domain (score of 2 or above), social domain (4 or above), and communication and social domains (7 or above) (33).

#### Collection of IQ Data

The HAWIK (Hamburg-Wechsler-Intelligenztest für Kinder) is the German version of the Wechsler Intelligence Scale for Children (e.g., WISC-IV) (34). In the ASD group, IQ scores had been regularly measured using HAWIK-IV and in some cases using the previous version HAWIK-III. The IQ of the control group had been measured with HAWIK-IV.

### Statistical Analysis

In accordance with the literature, established generic terms from family and developmental history were interpreted as individual families of hypotheses, representing sufficiently different research questions. In turn, items within the same generic term were corrected for multiple testing (35). In contrast, all data describing the sample (Tables 1, 2) were seen as sufficiently different units. Multiple *t*-tests of two-way ANOVA were performed using Prism software (GraphPad, San Diego, CA, USA). Correction for multiple testing was done by determining the false discovery rate (FDR) using the two-stage step-up method of Benjamini, Krieger, and Yekutieli. The *p*-value adjusted by FDR is expressed by the *q*-value. Differences were considered statistically significant if *q* < 0.05. Graphical artwork was created with Prism software. Graphs show *M*, standard error of the mean (*SEM*), and significant results from two-way ANOVA, corrected for multiple testing. The red color in the plotting area represents data about mothers of patients; blue color, data about fathers of patients; orange color, data about the whole family of patients; and green color, data about the patients themselves. One asterisk in a graph or column represents *q* < 0.05, two asterisks represent *q* < 0.01, and three asterisks represent *q* < 0.001. Tables show *M* and detailed results from two-way ANOVA. Detailed statistical results of graphs are deposited in Supplementary Table 2.

## Results

### Socioeconomic, Psychosocial, Psychiatric, and Medical History of Parents and Grandparents

In the following, items from the “family and developmental history questionnaire” were described when there was a statistical significant difference between adolescents with ASD and control persons after correction for multiple testing. Whenever a finding was initially significant, but showed no significance after correction for multiple testing, we described it as a tendency. Supplementary Table 1 shows questions from the “family and developmental history questionnaire,” the name of the respective item, allocation of the findings in figures and tables, and finally a description of the operationalization scheme. Detailed information of statistical analyses can be found in Supplementary Table 2.

**Figure 1:**
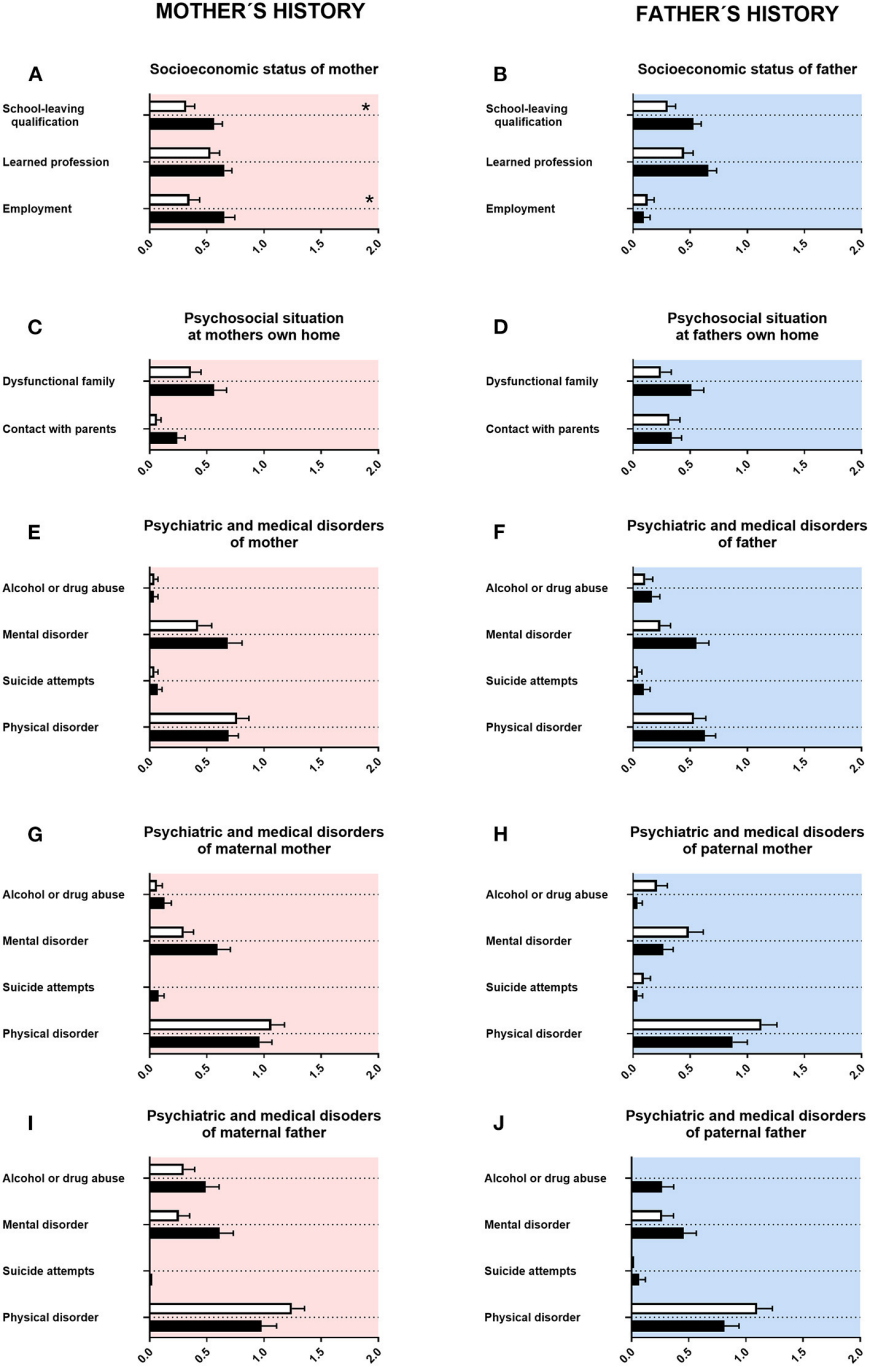
Family history. **(A,F)** The x-axes represent the level of educational or employment status, respectively. **(B–E, G–J)** The x-axes represent the level of psychiatric, psychosocial, or medical burden. Graphs show *M, SEM*, and results from two-way ANOVA, corrected for multiple testing. Black symbols represent autism spectrum disorder (ASD) patients, and white symbols, controls. Red color in the plotting area represents data about mothers of patients; and blue color, data about fathers of patients. *q < 0.05.

First, we assessed the history of parents and grandparents. For mothers of adolescents with ASD, lower school-leaving qualifications (associated with less years in school) were reported. For fathers of adolescents with ASD, there was a tendency for lower school-leaving qualifications (Figures 1A,B). Mothers of adolescents with ASD were more often in a part-time employment (Figure 1A). For fathers of adolescents with ASD, there was a tendency for less often achieving a university degree (Figure 1B). For mothers of adolescents with ASD, there was a tendency for fewer contact with their own parents (Figure 1C). For fathers of adolescents with ASD, a tendency for a higher rate of mental disorders was reported (Figure 1F). For maternal grandmothers and maternal grandfathers of adolescents with ASD, a tendency for a higher rate of mental disorders was reported (Figures 1G,I). For paternal grandfathers of adolescents with ASD, however, a tendency for more alcohol or drug consumption was reported (Figure 1J). For psychosocial situation at father's own home, psychiatric and medical disorders of mother and psychiatric and medical disorders of paternal mother, neither a significant difference nor a tendency was found (Figures 1D,E,H).

### Family Relationships, Conflicts in the Family, and Family Composition at Age 12–17

**Figure 2:**
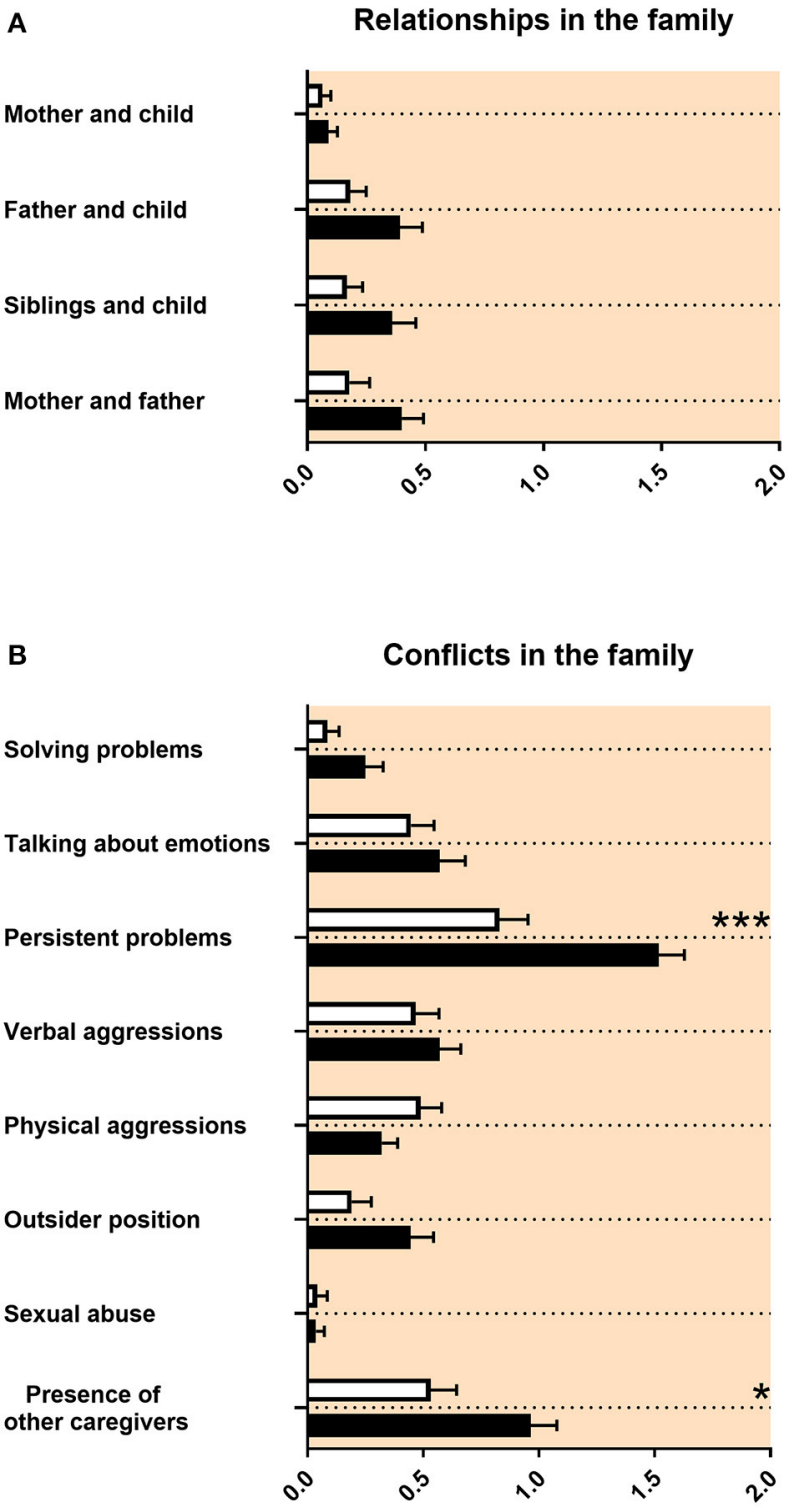
Family relationships and conflicts in the family. **(A,B)** The x-axes represent the level of psychosocial burden. Graphs show *M, SEM*, and results from two-way ANOVA, corrected for multiple testing. Black symbols represent autism spectrum disorder (ASD) patients; and white symbols, controls. Red color in the plotting area represents data about mothers of patients. *q < 0.05, and ***q < 0.001.

The quality of relationships with other family members was not impaired in adolescents with ASD (Figure 2A). Regarding conflicts in the family, there were more persisting problems reported for adolescents with ASD (Figure 2B). The composition of family members at the age 12–17 was different for adolescents with ASD compared with control persons. The number of persons living together in the family was reduced for adolescents with ASD. Mothers of adolescents with ASD reported less pregnancies and less childbirths (Table 3A). Children with ASD lived more often together with grandparents and less frequently together with biological siblings or half siblings (Table 3B).

**Table 3:** Developmental history—metric data.

	***M* (ASD)**	***M* (control)**	***t* ratio**	***df***	***p***	***q***
**A. Obstetrical history of the mother**						
Number of pregnancies (*n*)	2.76	3.68	2.73	100	0.007	0.015^*^
Number of childbirths (*n*)	2.25	2.77	2.19	100	0.031	0.031^*^
**B. Caregivers and family members living with the child at age 12–17**						
Number of biological parents (*n*)	1.66	1.70	0.43	101	0.671	0.542
Number of social parents (*n*)	0.16	0.13	0.44	101	0.663	0.542
Number of grandparents (*n*)	0.77	0.11	4.83	101	<0.001	<0.001^***^
Number of other adult caregivers (*n*)	0.07	0.00	1.88	101	0.063	0.088
Number of biological siblings (*n*)	0.07	1.34	7.38	101	<0.001	<0.001^***^
Number of half siblings (*n*)	0	0.17	2.43	101	0.017	0.034^*^
Number of social siblings (*n*)	0.04	0.11	1.11	101	0.268	0.280
Number of peers in youth welfare institutions (*n*)	0	0.02	1.09	101	0.277	0.280
Complete number of all persons living together with the child at the age of 12–17 (*n*)	2.79	3.51	2.83	101	0.006	0.015^*^
Only child family (*n*)	0.30	0.15	1.86	101	0.065	0.088
**C. Birth parameters**						
Age of mother at childbirth (years)	29.30 years	31.00 years	1.62	100	0.108	0.406
Age of father at childbirth (years)	33.2 years	33.4 years	0.18	100	0.860	0.869
Gestational age (weeks)	39.40 weeks	39.30 weeks	0.20	97	0.841	0.869
Birth weight (g)	3,571 g	3,512 g	0.51	100	0.613	0.869
Birth size (cm)	51.00 cm	51.20 g	0.44	100	0.656	0.869
APGAR 5 min (score)	9.60	9.40	1.60	92	0.115	0.406
APGAR 10 min (score)	9.80	9.70	0.71	91	0.479	0.869
**D. Markers of development (0–6 years)**						
Breastfeeding (months)	8.9 months	9.5 months	0.35	99	0.727	0.735
First free steps (months)	13.9 months	12.6 months	1.83	97	0.070	0.141
First words (months)	16.3 months	13.4 months	1.46	76	0.149	0.201
Nursery care—utilization (years)	0.7 years	1.0 years	1.60	101	0.114	0.184
Pre-school—utilization (years)	3.3 years	3.1 years	1.97	101	0.051	0.138
Right-handedness (%)	80%	95%	2.38	101	0.019	0.077
Ambidexterity (%)	0%	2%	1.09	101	0.277	0.320
Left-handedness (%)	19%	2%	2.84	101	0.005	0.044^*^
**E. School performance at primary school (6–10 years)**						
Repetition of one class (%)	17%	12%	0.71	101	0.482	0.162
Change of school (%)	25%	17%	0.98	101	0.330	0.133
Compensation for disadvantages (%)	70%	4%	9.21	100	<0.001	<0.001^***^
Regular primary school (%)	25%	97%	10.93	101	<0.001	<0.001^***^
Primary school with special needs setting (%)	44%	0%	6.10	101	<0.001	<0.001^***^
Special education in a special school (%)	28%	2%	3.81	101	<0.001^***^	<0.001^***^
**F. Transition into secondary school (10–17 years)**						
High school (%)	45%	68%	2.33	100	0.022	0.044^*^
Middle school, high performance (%)	29%	27%	0.16	100	0.875	0.883
Middle school, low performance (%)	14%	4%	1.75	100	0.083	0.112
Alternative or private school (%)	0%	0%				
Special education in secondary school (%)	14%	0%	2.80	100	0.006	0.025^*^

*Metric data assessed with the “family and developmental history questionnaire” represent milestones of development and other social and biological markers. The first experimental group (“ASD”) represents adolescents with a diagnosis of ASD and the other group (“control”) neurotypically developing adolescents. Participants of the study were the parents of the adolescents from these two groups. Columns show M and detailed results from two-way ANOVA, corrected for multiple testing*.

* *q < 0.05*,

** *q < 0.01, and*

*** *q < 0.001*.

*ASD, autism spectrum disorder*.

### Prenatal and Perinatal Maternal Psychosocial and Medical Complications and Maternal Stress

**Figure 3:**
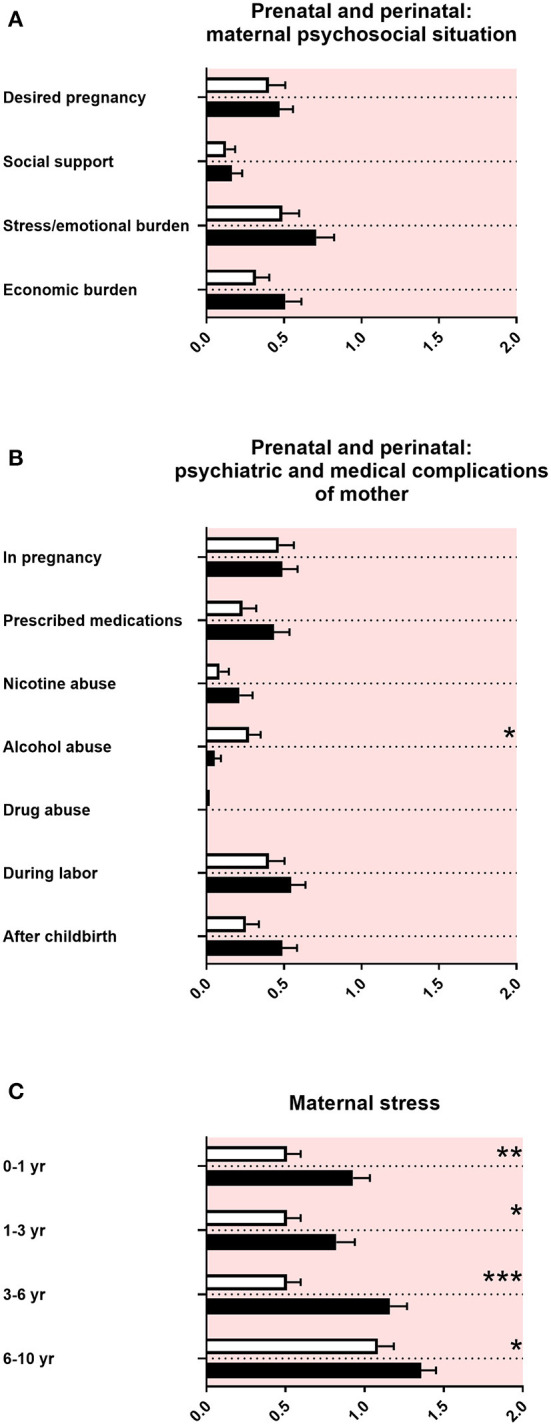
Prenatal and perinatal maternal psychosocial and medical complications and maternal stress. **(A)** The x-axis represents the level of psychosocial burden. **(B)** The x-axis represents the level of psychiatric and medical burden. **(C)** The x-axis represents the level of social and cognitive burden. Graphs show *M, SEM*, and results from two-way ANOVA, corrected for multiple testing. Black symbols represent autism spectrum disorder (ASD) patients; and white symbols, controls. Red color in the plotting area represents data about mothers of patients. *q < 0.05, **q < 0.01, and ***q < 0.001.

In the “family and developmental history questionnaire,” the maternal psychosocial situation during pregnancy with a baby later diagnosed with ASD was reported as unremarkable (Figure 3A). There were no differences in psychiatric and medical issues, except for less alcohol consumption of mothers pregnant with a baby later diagnosed with ASD (Figure 3B). There was increased maternal stress remembered from mothers of children with ASD from the first year of the child's life up to late childhood (Figure 3C).

### Prenatal and Perinatal History and Medical History of the Child

**Figure 4:**
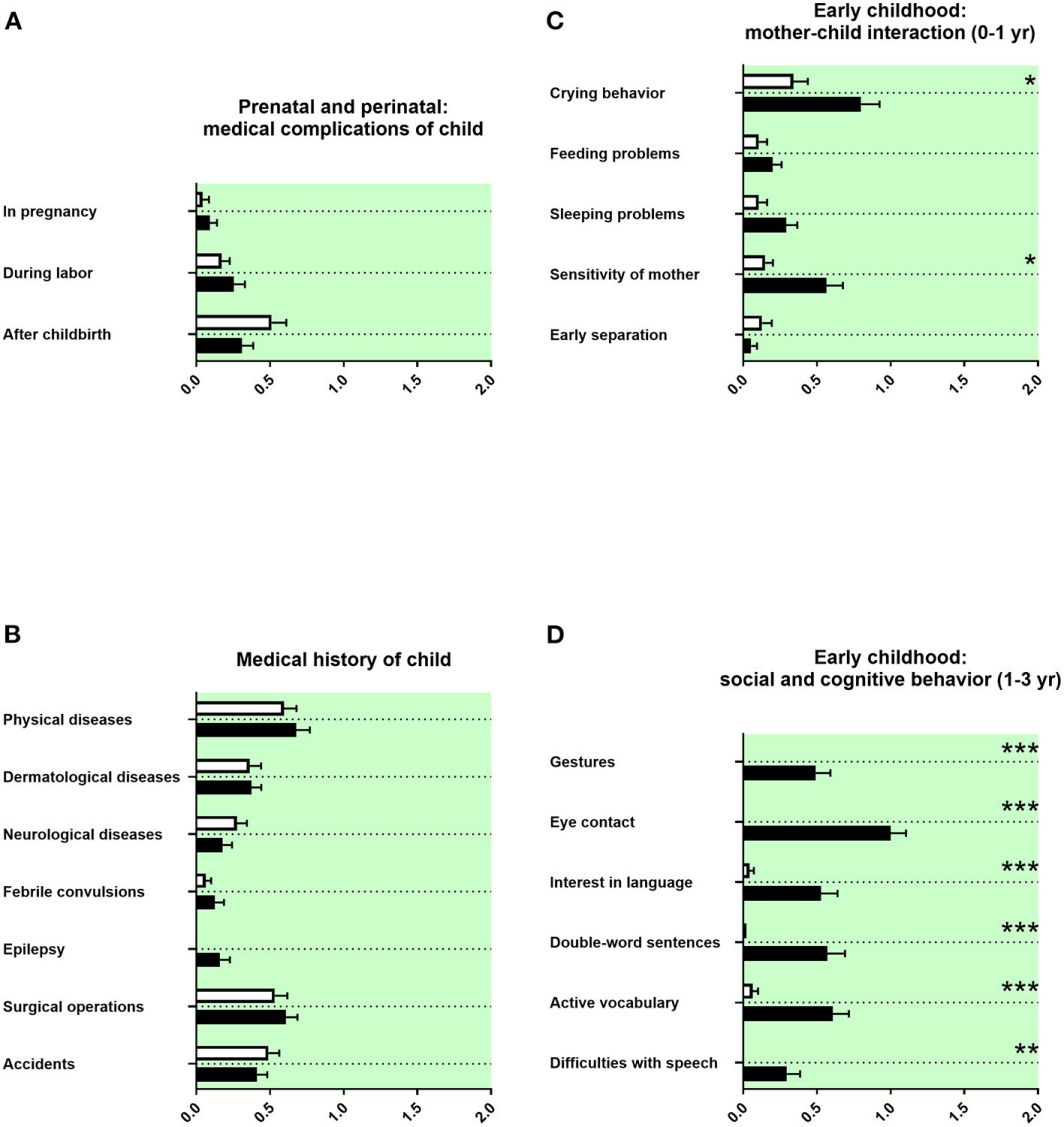
Prenatal and perinatal history, medical history of the child, and early childhood. **(A,B)** The x-axes represent the level of medical burden. **(C)** The x-axis represents the level of maladaptation between mother and child. **(D)** The x-axis represents the level of social and cognitive burden. Graphs show *M, SEM*, and results from two-way ANOVA, corrected for multiple testing. Black symbols represent autism spectrum disorder (ASD) patients; and white symbols, controls. Green color in the plotting area represents data about the patients themselves. *q < 0.05, **q < 0.01, and ***q < 0.001.

The prenatal and perinatal history and the medical history of the child was remembered as unremarkable (Figure 4A). A tendency for more epilepsy was reported in adolescents with ASD (Figure 4B). We found no differences in birth parameters (Table 3C).

### Early Childhood History

The behavior during early infancy is shaped by the interaction between mother and child. Parents of adolescents with ASD remembered increased crying behavior and less sensitivity of the mother (Figure 4C). Children with a diagnosis of ASD were reported using less gestures during communication at their first birthday, with less eye contact in their first year of life, and with reduced interest in language up to their first birthday. In addition, parents of children with ASD reported less double-word sentences and less active vocabulary (<50 words at the second birthday of the child) as well as more difficulties with speech (Figure 4D). Other markers of development during early childhood, as the first free steps or first words, were not remembered differently (Table 3D).

### Middle Childhood History

**Figure 5:**
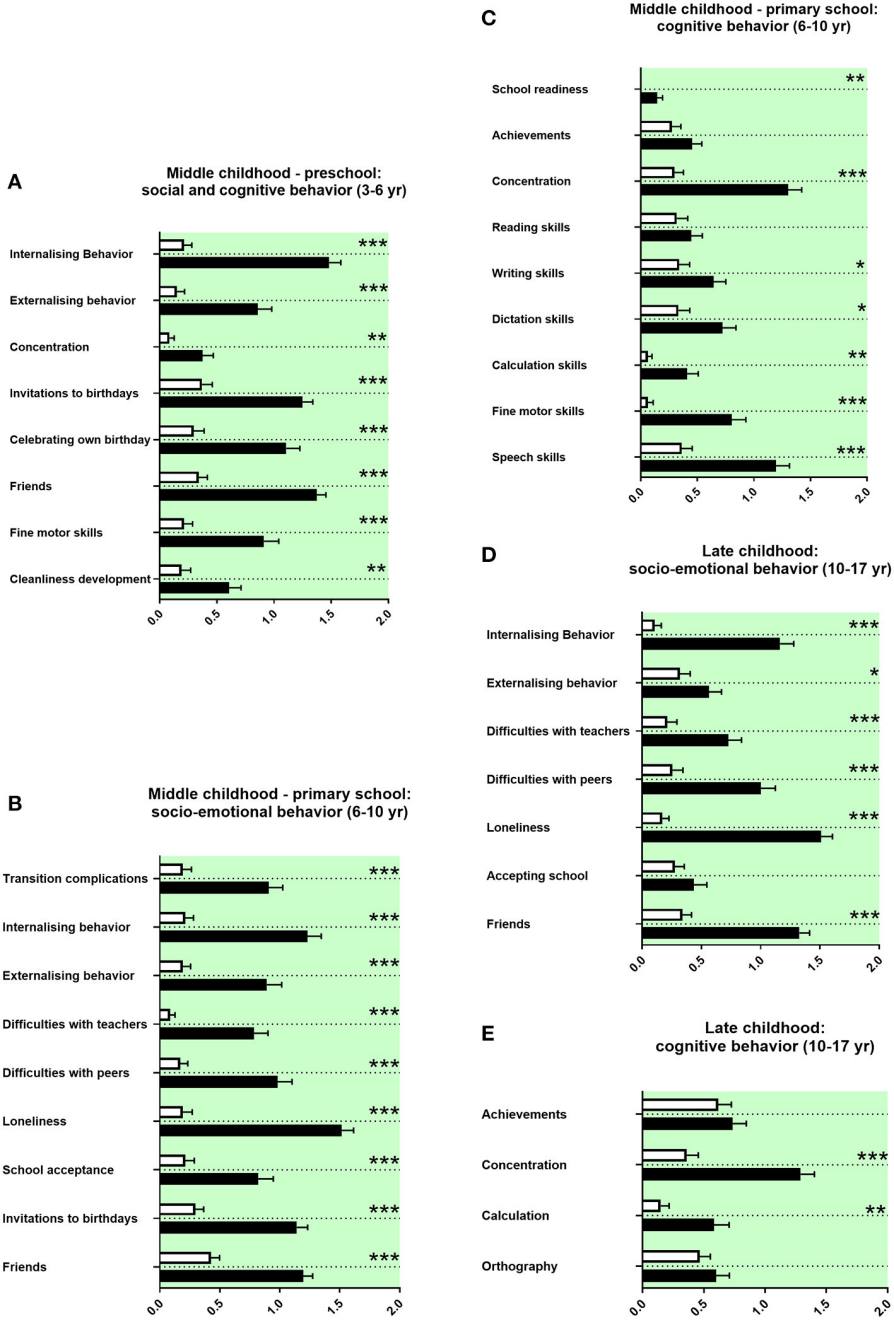
Middle and late childhood history. **(A–E)** The x-axes represent the level of socio-emotional or cognitive burden. Graphs show *M, SEM*, and results from two-way ANOVA, corrected for multiple testing. Black symbols represent autism spectrum disorder (ASD) patients; and white symbols, controls. Green color in the plotting area represents data about the patients themselves. *q < 0.05, **q < 0.01, and ***q < 0.001.

We assessed the socio-emotional and cognitive behaviors of adolescents with ASD during preschool and primary school. Parents of children with ASD reported at both preschool and primary school age more internalizing and externalizing child behavior, less concentration of the child (Figures 5A,C), and also fewer invitations to others' birthday parties, less celebrations of own birthdays, and less firm friends (Figures 5A,B). Parents of adolescents with ASD reported more complications for the child in transition to primary school, more difficulties with teachers, more difficulties with peers, more loneliness, and more school avoidance (Figure 5B). They reported more often a school start 1 year later and reduced writing skills, reduced dictation skills, reduced calculation skills, reduced fine motor skills at preschool age, and at primary school age reduced speech skills (Figures 5A,C). Concerning school performances, adolescents with ASD received more often compensations for disadvantages at primary school. Fewer adolescents with ASD went to regular primary schools; most of them received a special needs setting in primary school or a school placement for special education (Table 3E).

### Late Childhood History

Parents of adolescents with ASD reported more internalizing and more externalizing behaviors in late childhood. They also reported more difficulties with teachers and peers, more loneliness, and less firm friends (Figure 5D). They reported less concentration in school and more difficulties with calculation (Figure 5E). Furthermore, adolescents with ASD attended less often high school and received more often a placement in special education settings in secondary school (Table 3F).

## Discussion

Modeling the initial detection setting of ASD in adolescents performed by regular MHPs, we assessed here family and developmental history using the “family and developmental history questionnaire” as previously described for ADHD (28). Adolescents with ASD expressed a profile of family and developmental history items that significantly differed from adolescent control persons. Within these findings, the majority of significant items matched with descriptions in the ASD literature.

### Comparison of Study Findings With the Autism Spectrum Disorder Literature

#### Socioeconomic, Psychosocial, Psychiatric, and Medical History of Parents and Grandparents

Our data showed for mothers of adolescents with ASD lower school-leaving qualifications (associated with less years in school). Mothers of adolescents with ASD were more often in a part-time employment (Figure 1A). For fathers of adolescents with ASD, there was a tendency for lower school-leaving qualifications, and they had less often a university degree (Figure 1B). This is in agreement with some studies describing lower socioeconomic status for both mothers and fathers of adolescents with ASD, including employment status. However, other studies described higher school and academic certifications (36–42).

For mothers of adolescents with ASD, we found in our data a tendency for fewer contact with their own parents (Figure 1C). In qualitative studies, increased stress and challenges in the interaction with their grandchildren were reported for grandmothers of adolescents with ASD, thus reporting some similarities (43–46). In our study, we found for fathers of adolescents with ASD a tendency for a higher rate of mental disorders (Figure 1F). This is in line with the literature, as for both mothers and fathers of children with ASD a higher rate of mental disorders was reported (47–51).

For maternal grandmothers and maternal grandfathers of adolescents with ASD, we found in our data a tendency for a higher rate of mental disorders (Figures 1G,I). For paternal grandfathers of adolescents with ASD, however, a tendency for more alcohol or drug consumption was observed (Figure 1J). For psychiatric disorders in general, a higher rate of mental disorders in grandparents of the index patient can be found in the literature. Some of those reports are specifically for ASD (43, 49, 52, 53).

#### Family Relationships, Conflicts in the Family, and Family Composition at Age 12–17

Our study revealed that the quality of relationships with other family members was not impaired in adolescents with ASD (Figure 2A). Regarding conflicts in the family, there were more persisting problems reported for adolescents with ASD (Figure 2B). This is in line with the ASD literature, as individual relationships in families of adolescents with ASD were not described as markedly impaired. However, families reported lower functioning of the family and decreased quality of life (54, 55). We found in our data that mothers of adolescents with ASD reported less pregnancies and less childbirths (Table 3A). It was discussed that less biological siblings and less half siblings in families with an adolescent with ASD could be an effect of “reproductive stoppage” in the family planning process of the patient's parents (56, 57). In our data, the number of persons living together in the family was reduced for adolescents with ASD during the age of 12–17, including adolescents with ASD living less frequently together with biological siblings or half siblings (Table 3B). This appears to be related to the reproductive behavior of the parents, too. Finally, in our findings, children with ASD lived more often together with grandparents (Table 3B). For this last finding, we did not find a description in the literature.

#### Prenatal and Perinatal Maternal Psychosocial and Medical Complications and Maternal Stress

In our study, we did not find increased prenatal and perinatal psychosocial, psychiatric, and medical complications in mothers of children with ASD, using the “family and developmental history questionnaire” (Figures 3A,B). By contrast, there is a large body of literature on small but significant prenatal and perinatal risk factors found in epidemiological cohorts, such as maternal infections and perinatal complications (58, 59). An explanation for the discrepancy between our data and the findings in the literature could be the exclusion of adolescents with ASD with intelligence deficiency in our study, which are associated with a higher burden of disease. In addition, large epidemiological studies were often based on serological data, patient files, or health registers, whereas the “family and developmental history questionnaire” solely relies on the memory of non-experts in terms of medical knowledge. Finally, a difference between large cohorts and our study could result from the number of participants and therefore different statistical power.

In accordance with the literature, our data showed an increased maternal stress from toddler age until middle childhood in families with an individual with ASD (Figure 3C) (43, 60–62).

#### Prenatal and Perinatal History and Medical History of the Child

In our data, a tendency for more epilepsy in adolescents with ASD was reported (Figure 4B). In the literature, epilepsy is well-known to be associated with ASD (63). We found in our data set no differences in birth parameters (Table 3C). By contrast, several prenatal and perinatal medical complications, such as fetal distress, birth asphyxia, and neonatal jaundice, were associated in the literature with a higher risk of ASD (40, 49). As we have discussed above, this discrepancy could be explained by the different study designs.

#### Early Childhood History

In our study, parents of adolescents with ASD remembered increased crying behavior and less sensitivity of the mother (Figure 4C). In the literature, behavioral problems in early infancy, such as crying behavior, feeding problems, and sleeping problems, have all been linked with increased psychopathology (64–67). Altered responsiveness of mothers of children with ASD has been described (68).

In our data, children with a diagnosis of ASD were reported using less gestures during communication at their first birthday, with less eye contact in their first year of life, and with reduced interest in language up to their first birthday. In addition, parents of children with ASD reported less double-word sentences and less active vocabulary (<50 words at the second birthday of the child) as well as more difficulties with speech (Figure 4D). In strong agreement, difficulties in social and communicative development and behavior during early childhood are described in the literature as part of the ASD symptomatology (67, 69–71). Surprisingly, established neuropediatric markers of development during early childhood, as the first free steps or first words stated in months, were not remembered differently by parents in our investigation (Table 3D). An explanation could be that in the clinical diagnostic interview, especially qualitative content seems to be better remembered than quantitative content (72).

#### Middle Childhood History

In our study, parents of children with ASD reported at both preschool and primary school more internalizing and externalizing child behavior, less concentration of the child (Figures 5A,C), and also fewer invitations to others' birthday parties, less celebrations of own birthdays, and less firm friends (Figures 5A,B). In line with these study findings, various difficulties during preschool in social and cognitive behaviors have been described in the literature, internalizing and externalizing behaviors, and concentration difficulties (73). In line with the literature, our data show more withdrawal behaviors and less social relatedness for children with ASD (Figure 5B) (73). Our data set revealed reduced fine motor skills and a delayed cleanliness development. Impairments in movement development and cleanliness development are described (74, 75).

For children with ASD attending pre-school, our data show difficulties with teachers at school. In the literature, missing resources and overstrained staff teaching children with ASD were reported (76). Our data show in line with the literature less right-handedness and more left-handedness in children with ASD (77). In our study, parents of adolescents with ASD reported more complications for the child in transition to primary school, more internalizing behavior, more externalizing behavior, more difficulties with teachers and peers, more loneliness, and less school acceptance, less firm friends, and less invitations to birthdays (Figure 5B). In accordance with these findings, difficulties in socio-emotional behavior are well-documented as part of the core ASD symptomatology at primary school age. These literature findings include internalizing and externalizing behaviors; difficulties with teachers, peers, and friendships; more withdrawal behaviors; transition complications; and school avoidance (78–83). The parents in our study reported reduced school readiness, reduced concentration in lessons and reduced writing skills, reduced dictation skills, reduced calculation skills, reduced fine motor skills, and reduced speech skills (Figure 5C).

In agreement with our findings, many impairments in cognitive behavior were reported in children with ASD at primary school age. These include delayed start of school, impaired concentration, altered literacy acquisition, and reduced mathematical competencies (78, 84–86). Impaired movement development and impaired speech skills are also well-known in children with ASD (87, 88). In our set of data, adolescents with ASD received more often compensations for disadvantages at primary school. Fewer adolescents with ASD went to regular primary schools; most of them received a special needs setting in primary school or a special education (Table 3E). In agreement, the literature shows that children with ASD were more often placed in special needs settings (89, 90).

#### Late Childhood History

As for middle childhood, parents of adolescents with ASD reported more internalizing and more externalizing behaviors in late childhood. They also reported more difficulties with teachers and peers, more loneliness, and less firm friends (Figure 5D). They reported less concentration in school and more difficulties with calculation (Figure 5E). Furthermore, adolescents with ASD attended less often high school and received more often a special education in secondary school (Table 3F). In the literature on ASD, problems in socio-emotional behavior persist into late childhood, including internalizing behaviors, difficulties with peers, more loneliness, and problems with friendships (79, 91–93). In this line, cognitive problems are described for late childhood, including school achievements, concentration, calculation, and spelling (94, 95). In late childhood, ASD patients were also more often placed in special needs settings (89, 90).

#### Summary of Comparison Between Study and Literature Findings

Most items of the “family and developmental history questionnaire” showing significant differences or tendencies between adolescents with ASD and control persons are in accordance with descriptions in the literature on ASD. There were some exceptions. These exceptions appear to be related to the technique of the clinical diagnostic interview and to different designs between studies reported in the literature and ours described here.

### Profile of Significant Items in the Group of Adolescents With Autism Spectrum Disorder

Many memories from parents of adolescents with ASD significantly differed in the “family and developmental history questionnaire” from memories of parents of control persons. ASD-specific instruments like ADI-R and SRS, also assessing relevant aspects of family and developmental history, do this from a clinician's viewpoint at an ASD-specialized center or team, when ASD is already suspected and the patient referred to investigate this hypothesis. By contrast, here, we took a look at family and developmental history from the viewpoint of MHPs in their general practice, non-specific and without the hypothesis of ASD. To our knowledge, we describe here for the first time with *quantitative empiricism* an analysis of a data set of family and developmental history, modeling the scenario as it is obtained by general MHPs using the regular clinical diagnostic interview, in distinction to specialized ASD centers or teams using ASD-specific tools such as ADI-R, SRS, or AQ.

The memory of parents of adolescents with ASD revealed strong significance for early signs of ASD, notably in social and cognitive behavior at 1–3 years of age (Figure 4D). Less gestures, impaired eye contact, and impaired language development are markers of the core symptomatology of ASD. On first glance, it may appear a trivial finding that those items most linked with ASD showed strong significance. However, it could show that parents do remember these issues well-even after long time. Alternatively, the memory of parents of adolescents with ASD could have been biased by psychoeducation and lay knowledge.

Items, especially from later developmental history, seemed to show more significant differences between adolescents with ASD and control persons than those from family history. A possible explanation is that memories of more recent events are more precise than those of past events. For example, the ADI-R performed with caregivers of adult individuals with ASD and normal intelligence appeared less reliable than an interview with parents of children (96). Another explanation could be that interviewed parents were reluctant to report subjectively shameful information about other family members.

In addition, the findings suggest that a broad selection of items in combination with thoroughness of the clinical diagnostic interview procedure is important for the generation of diagnostic hypotheses, here ASD. The collection of items, “family and developmental history questionnaire,” was first used to study a similar scenario during initial detection of ADHD (28). We used here the same non-specific collection of items in the context of ASD. Thus, a well-selected and thoroughly performed clinical diagnostic interview, as the unspecific diagnostic instrument used by MHPs, seems to be useful and supportive to generate a specific hypothesis of ASD. As described above, the hypothesis of ASD can then be confirmed or discarded at a specialized center or team.

### Relevance of the Clinical Diagnostic Interview of Family and Developmental History for Generation of an Autism Spectrum Disorder Hypothesis in Adolescents

We could show that a significant profile of family and developmental history in the context of ASD can be obtained with an established clinical method in adolescents. The clinical diagnostic interview as a face-to-face approach is commonly used in different clinical settings, in particular general MHP settings, to survey family and developmental history. It facilitates generating a relationship of trust with parents and patients, catching a personal impression of the family, and clarifying statements and is time-economic. Moreover, the clinical diagnostic interview supports the generation of working hypotheses by the experienced clinician, because information is directly interpreted. In contrast, rating scales as pen-and-pencil methods with inflexible structure interfere with relationship building, and given information is primarily a collection of symptoms. Therefore, rating scale information is an important complement of the clinical diagnostic interview, but cannot replace it.

A population-based study from the United Kingdom proposed that out of five children with the clinical syndrome of ASD at primary school age, only three receive the diagnosis of ASD, and two are not detected (97). A significant proportion of these undetected children reach adolescence or even adulthood without a diagnostic assessment by an MHP that would lead to referral to a specialized center for autism (98). Moreover, there is an important interval from first concerns to final diagnosis of ASD in adolescents and adults. For high-functioning autism in adults, it was reported to take about 2 years to confirm the diagnosis (99). While the diagnostic process of early detection and generating the hypothesis of autism in childhood appears well-established, a structure resulting in referral to a specialized ASD center or team seems to only slowly emerge for adults, presumably also adolescents (100). These problems are also described in the current literature, suggesting their persistence (10, 17).

To achieve a time-effective diagnosis, which results into sooner treatment, the transition from first consultation of the MHP to confirmation of the diagnosis should be as short as possible. The quality of generating the initial hypothesis depends on the quality of the clinical assessment, including the clinical diagnostic interview. Reasons for the still reduced quality were identified as limited knowledge about ASD, the common referral pathways, and finally the use of insufficient diagnostic tools (101, 102). Given that the clinical diagnostic interview, skillfully and thoroughly performed by a clinician face to face, provides guidance throughout the diagnostic process may help to decrease time between initial detection and confirmation of ASD diagnosis as mentioned above.

Finally, a state-of-the-art psychopathological examination including a full clinical diagnostic interview requires substantial time, to our experience rarely <60 min. We acknowledge that clinicians, especially MHPs in general mental healthcare settings, are often short of time. However, reducing quality of the initial diagnostic process increases the likelihood of misdiagnosis or delay of diagnosis.

### Limitations of the Study

The limitations our study are non-blinded student interviewers, only male adolescents, localized samples, and a limited number of participants. Our study was an exploratory study and equipped with limited resources. Future research activities are necessary to study these conventional diagnostic methods and scenarios modeling the phase of generating an ASD hypothesis in adolescents and adults. These future research activities should include the comparison of an ASD cohort with other psychiatric disorders, for example, social anxiety, conduct, and oppositional-defiant disorders and ADHD, in order to investigate differential diagnoses as well as the impact of general psychopathology. An improved study design should also include several centers, blinded interviewers, adolescents of all genders, board-certified interviewers, and significantly more statistical power. Such an improved design could include additional items in order to identify an optimized set of family and developmental history questions for both identification of specific psychiatric diagnoses and discrimination between differential diagnoses. The selection of items with most specificity and sensitivity could establish an optimized tool for clinical practice, regarding family and developmental history.

## Conclusion

Our findings for adolescents with ASD support importance and feasibility of the family and developmental history in the initial diagnostic process (28, 103, 104). In particular, a thorough interview of family and developmental history in individual patients seems to be of relevance for making the hypothesis of ASD. It may help to decrease the interval until the individual is referred to an ASD specialized center to confirm the diagnosis.

## Data Availability Statement

The original contributions presented in the study are included in the article/Supplementary Material, further inquiries can be directed to the corresponding author/s.

## Ethics Statement

The studies involving human participants were reviewed and approved by Ethics Committee of the University Hospital Carl Gustav Carus (reference number EK 295072016). Written informed consent to participate in this study was provided by the participants' legal guardian/next of kin.

## Author Contributions

JW collected the data, interpreted the results, and wrote the paper. CC collected the data and wrote the paper. KA supervised the clinical specialist diagnosis of ASD patients. VR wrote the paper. RW designed the study, supervised the collection of data, interpreted the results, and wrote the paper. All authors contributed to the article and approved the submitted version.

## Conflict of Interest

The authors declare that the research was conducted in the absence of any commercial or financial relationships that could be construed as a potential conflict of interest.

## Publisher's Note

All claims expressed in this article are solely those of the authors and do not necessarily represent those of their affiliated organizations, or those of the publisher, the editors and the reviewers. Any product that may be evaluated in this article, or claim that may be made by its manufacturer, is not guaranteed or endorsed by the publisher.

## Supplementary Material

Supplementary Table 1 “Family and developmental history questionnaire.” The questions asked for each individual item are displayed. For items with non-metric verbal answers, the mode of operationalization by the research team is indicated. For items with metric answers, the measuring unit is indicated.

Supplementary Table 2 Details of statistical analyses shown in Figures 1–5. Columns show *M, SEM* and detailed results from two-way ANOVA, corrected for multiple testing. ^*^*q* < 0.05, ^**^*q* < 0.01 and ^***^*q* < 0.001.
